# Sex-Specific Effects of Early-Life Unpredictability on Hippocampal and Amygdala Responses to Novelty in Adolescents

**DOI:** 10.1016/j.bpsgos.2025.100561

**Published:** 2025-07-07

**Authors:** Elysia Poggi Davis, Bianca T. Leonard, Robert J. Jirsaraie, David B. Keator, Steven L. Small, Curt A. Sandman, Victoria B. Risbrough, Hal S. Stern, Laura M. Glynn, Michael A. Yassa, Tallie Z. Baram, Jerod M. Rasmussen

**Affiliations:** a Department of Psychology, University of Denver, Denver, Colorado; b Department of Pediatrics, University of California, Irvine, California; c Center for the Neurobiology of Learning and Memory, University of California, Irvine, California; d Department of Neurobiology and Behavior, University of California, Irvine, California; e Division of Computational and Data Sciences, Washington University in St. Louis, St. Louis, Missouri; f Department of Psychiatry and Human Behavior, University of California, Irvine, California; g Change Your Brain Change Your Life Foundation, Costa Mesa, California; h Amen Clinics, Costa Mesa, California; i School of Behavioral and Brain Sciences, University of Texas at Dallas, Dallas, Texas; j Centre of Excellence for Stress and Mental Health, VA San Diego Healthcare System, La Jolla, California; k Department of Psychiatry, University of California San Diego, La Jolla, California; l Department of Statistics, University of California, Irvine, Irvine, California; m Department of Psychology, Chapman University, Orange, California; n Department of Anatomy and Neurobiology, University of California, Irvine, California

**Keywords:** Early-life adversity, Functional magnetic resonance imaging (fMRI), Limbic system, Novelty, Sex differences, Unpredictability

## Abstract

**Background:**

Unpredictable childhood experiences are an understudied form of early-life adversity that impact neurodevelopment. The neurobiological processes by which exposure to early-life unpredictability impact development and vulnerability to psychopathology remain poorly understood. In the current study, we investigated the sex-specific consequences of early-life unpredictability on the limbic network, focusing on the hippocampus and the amygdala.

**Methods:**

Participants included 150 youths (54% female). Early-life unpredictability was assessed using the Questionnaire of Unpredictability in Childhood (QUIC). Participants engaged in 1 or more task–functional magnetic resonance imaging scans between the ages of 8 and 17 (223 total observations) measuring blood oxygen level–dependent (BOLD) responses to novel and familiar scenes.

**Results:**

Exposure to early-life unpredictability was associated with BOLD contrast (novel vs. familiar) in a sex-specific manner. For boys, but not girls, higher QUIC scores were associated with lower BOLD activation in response to novel versus familiar stimuli in the hippocampal head and amygdala. Secondary psychophysiological interaction analyses revealed complementary sex-specific associations between QUIC scores and condition-specific functional connectivity between the right and left amygdala, as well as between the right amygdala and hippocampus bilaterally.

**Conclusions:**

Exposure to unpredictability in early life has persistent implications for the functional operations of limbic circuits. Importantly, consistent with emerging experimental animal and human studies, the consequences of early-life unpredictability differ for boys and girls. Furthermore, impacts of early-life unpredictability were independent of other risk factors including lower household income and negative life events, indicating distinct consequences of early-life unpredictability beyond more commonly studied types of early-life adversity.

## Plain Language Summary

Unpredictable environments during early life can impact the way a child’s brain develops, particularly in brain regions related to stress, emotion, and memory. In this study, we observed 150 youths and found that early-life unpredictability affected regional brain activity in a sex-specific manner. Males with higher unpredictability scores showed lower activation in the hippocampus and amygdala when viewing novel scenes, while females did not. In addition to activity, unpredictability influenced brain connectivity between the hippocampus and amygdala differently in males and females. These observations were independent of other adversity factors, highlighting unpredictability as a unique influence on brain development.

A converging body of research across species demonstrates that early-life adversity (ELA) disrupts brain development and increases risk for mental health disorders (1, 2, 3, 4, 5). Over several decades, studies have investigated unique subtypes of ELA (e.g., deprivation vs. threat) and their role in the emergence of cognitive, emotional, and psychiatric outcomes (6, 7, 8, 9, 10). More recently, advances in neuroimaging have enabled the linking of behavioral changes associated with ELA to altered brain structure and function across development (11, 12, 13, 14, 15, 16, 17, 18, 19, 20).

While ELA and its subtypes are now well established as key contributors to outcome variance at the population level, their capacity to predict cognitive and emotional outcomes at the individual level remains limited (21). This has prompted the search for novel types of ELA that are not fully captured by the tools commonly used to characterize ELA. In response, we and others have examined unpredictability of parental and environmental signals as a distinct and potentially important form of ELA (22, 23, 24, 25, 26, 27, 28, 29, 30, 31). These efforts have identified significant cross-species effects of unpredictability, measured through experimental paradigms in animals and observations and questionnaires in humans, across several domains, including executive function (24, 25, 26,32), memory (33, 34, 35), behavioral problems (36), and hedonic behaviors (37, 38, 39).

Recent evidence suggests that the exploration of and responses to novelty are essential for healthy neurodevelopment, including learning and memory (40). Several groups have demonstrated that novelty processing is responsive to various forms of ELA exposure including physical abuse in nonhuman primates (41), childhood maltreatment in adults (42), and neglect (a common form of maltreatment) in adolescents (43). Moreover, compelling evidence indicates that exposure to early-life unpredictability alters child exploration behaviors, including exploration of novelty (40). Importantly, these behavioral phenotypes are consistent with observed associations between early-life unpredictability and anxiety symptoms (22,44, 45, 46, 47). Therefore, in the current study, we investigated how unpredictability influences children’s interactions with novel stimuli.

Processing of novelty engages several regions in the limbic network, including the hippocampus (48) and amygdala (amyg) (49). Thus, it is not surprising that these 2 regions are both among the most vulnerable to the effects of ELA (50, 51, 52, 53), including unpredictability, and are implicated in a host of mental health disorders including anxiety, depression, and posttraumatic stress disorder (54,55). In addition, there exists a large historical body of evidence to provide context for the effects of stress specifically on these 2 structures (56,57). Given this literature, and considering multiple comparisons (58) and expected effect sizes in brain-behavior/exposure relationships (59), here we probe the effects of exposure to early-life unpredictability on the neural response to novel stimuli, focusing on the hippocampus and amygdala.

While ELA is a risk factor for mental illnesses in both males (M) and females (F), rates and symptoms differ quantitatively and qualitatively by sex. For example, in adolescents, rates of major depressive episodes are higher in females, and patterns of initiation of substance use and abuse differ among males and females (60, 61, 62). Although sex differences in mental illness and sex-modulated changes in brain organization are often studied in the context of adolescence and puberty, the male and female brain differ already during the perinatal period (63, 64, 65, 66). A growing body of research with rodents, nonhuman primates, and humans suggest that males and females respond differently to early environmental signals beginning as early as conception and persisting throughout childhood (20,67, 68, 69, 70). In mice, encoding of unpredictable signals varies by sex as early as postnatal day 6 (71). Furthermore, mechanistic rodent studies have characterized the sex-specific organization of neural circuitry subserving cognitive and emotional functions resulting in sexually dimorphic behavioral phenotypes (e.g., increased propensity for substance seeking in females and increased anhedonia in males) in response to unpredictability (37, 38, 39,72,73). However, the results of studies that have examined sex-specific associations between exposure to early-life unpredictability and phenotypic (24,74,75) and neuroimaging (76) outcomes in humans have been mixed. For example, one study found that unpredictability was associated with frontolimbic structural connectivity among females but not males; however, the interaction failed to reach significance at the *p* < .05 threshold (*p* = .086) (76). Nonetheless, the motivation for sex-specific investigation is well supported given the collective evidence that 1) ELA (9) and unpredictability are risk factors for mental illness (24); 2) rates and symptoms of mental illness differ by sex (53,60); 3) sexually dimorphic brain development patterns emerge early and are observable via neuroimaging (20,77); and 4) preclinical models support sex-specific ELA programming, including unpredictability (71). Therefore, we tested the hypothesis that the effects of unpredictability on brain development differ by sex.

To test the hypothesis that early-life unpredictability affects hippocampal and amygdalar responses to novelty in a sex-dependent manner, we conducted a longitudinal functional magnetic resonance imaging (fMRI) study based on the response to novel relative to familiar stimuli in 150 participants with a total of 223 imaging sessions across childhood and adolescence (ages 8–17 years). Early-life unpredictability was characterized using the well-validated Questionnaire of Unpredictability in Childhood (QUIC). Primary analyses considered sex-specific associations between the QUIC and novel relative to familiar blood oxygen level–dependent (BOLD) contrasts using mixed-effects models. This task and others like it, in the context of hippocampal/amygdala activation, have been validated in detail (78, 79, 80), and developmental changes in this cohort have been described previously (81). To test whether associations were specific to unpredictability, we conducted specificity analyses covarying for other commonly studied forms of ELA. Finally, because the amygdala and hippocampus function as part of a broader circuit, we conducted a secondary analysis to examine their context-dependent (novel vs. familiar) functional connectivity using a traditional psychophysiological interaction (PPI) model.

## Methods and Materials

### Study Overview

**Figure 1:**
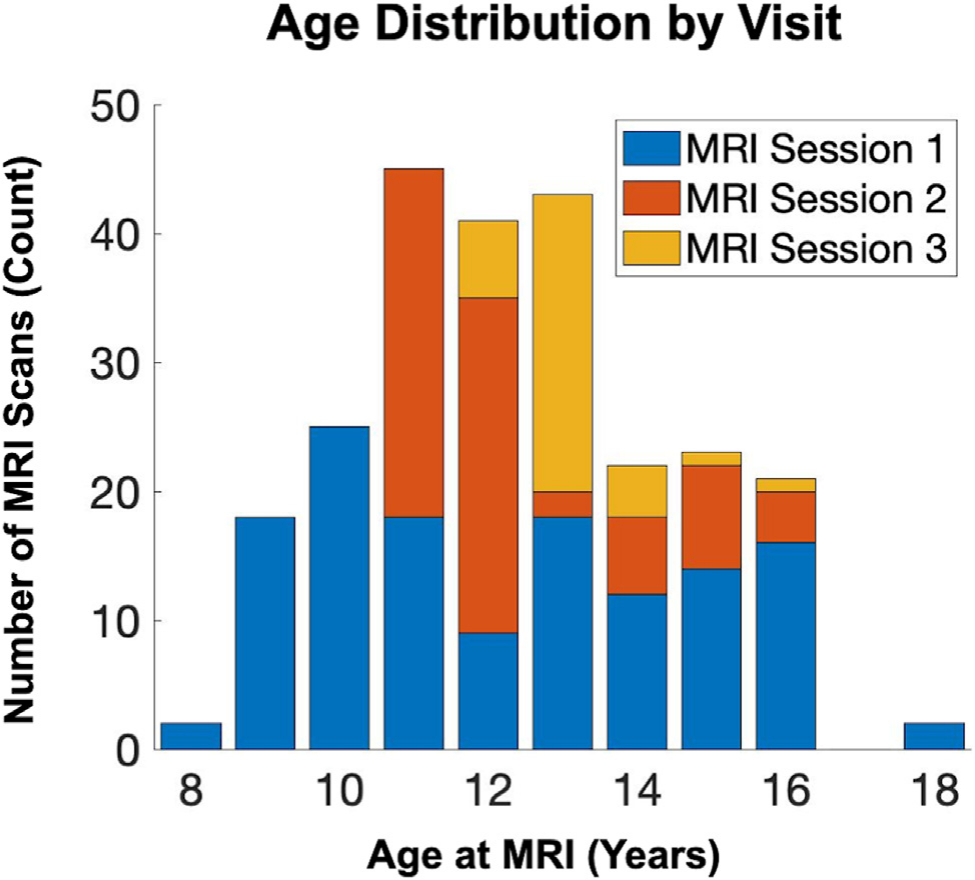
Age distribution by visit. Age distribution (range 8.2–17.7 years) across imaging sessions/visits is shown. MRI, magnetic resonance imaging.

Participants included 150 youths (54% female) participating in a longitudinal study of maternal and child health (22). Participants completed functional scanning between the ages of 8 and 17 years (mean ± SD age = 12.5 ± 2.0) (Figure 1). The study protocol was approved by the institutional review boards at the University of California Irvine and Chapman University, and written informed consent was obtained from the mothers, and assent was obtained from the children. See Table 1 for demographic information.

**Table 1:** Participant Demographic Characteristics *(N* = 150)

Characteristics	Values
Child Race and Ethnicity	
Asian	7.1%
Black	4.2%
Hispanic or Latinx	36.9%
Multiethnic	14.9%
Non-Hispanic White	36.9%
Child Age at Scan, Years	12.5 ± 2.0
Maternal Education	
High school or less	13.9%
Some college or associates degree	39.4%
4-year college degree	30.9%
Graduate degree	15.8%
Cohabitation Status, Cohabiting	86.6%
Median Income-to-Needs Ratio	4.11
Child Sex at Birth, Female	53.6%

Values are presented as % or mean ± SD unless otherwise specified.

### Measurement of Early-Life Unpredictability

Early-life unpredictability was characterized using the validated 38-item version of the QUIC (45,75,82). In brief, QUIC items assess unpredictability in the social, emotional, and physical domains that participants endorse as applying to their life prior to their current age. QUIC scores range from 0 to 38, with a higher score indicating greater exposure to unpredictability. The QUIC scores used here were based on self-report and acquired at the first visit (mean age = 12.2 ± 1.3 years). Additional details on the QUIC are included in Supplement, section S1.

### Measurement of BOLD Response to Novel Relative to Familiar Stimuli

**Figure 2:**
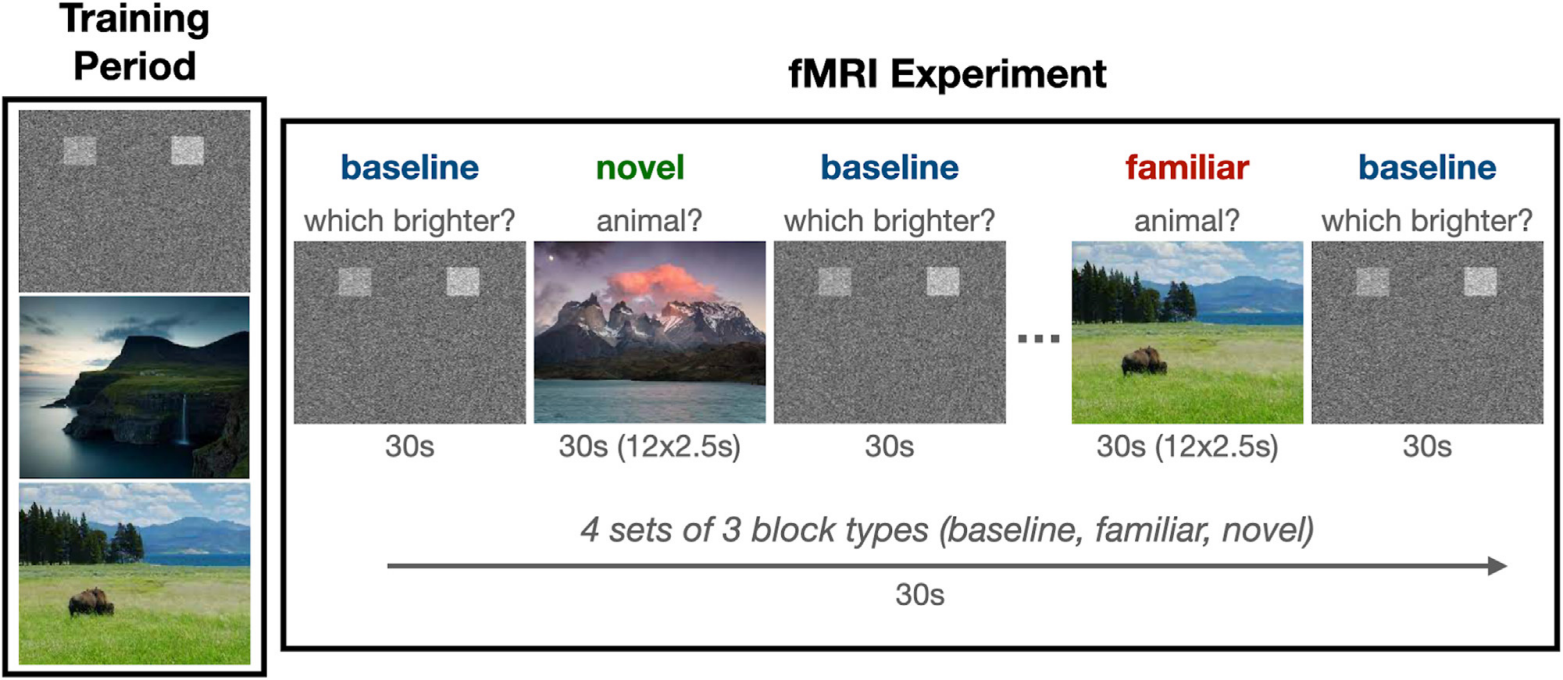
Task paradigm. Participants underwent an initial training session prior to imaging to familiarize themselves with the task and outdoor scenes. Novel and familiar outdoor scenes were presented in a block design. fMRI, functional magnetic resonance imaging.

The neural response to novel relative to familiar scenes was characterized using a task of incidental memory encoding (Figure 2). This task was designed to be child-friendly and consisted of landscape pictures (selected from the National Geographic library) that were either familiar (seen during a training period before the fMRI session) or novel (not seen during the training period). Children were instructed to press the left (index finger, dominant hand) button when an animal was in the scene and the right (middle finger, dominant hand) button when no animal was present. An additional baseline task was included that required participants to discriminate between 2 noise boxes of differing brightness against a noise background. The intensity of the boxes was randomized, and participants were instructed to press the button matching the brightest opacity. Four sets of three 30-second block types were presented in random order and counterbalanced across participants. Within blocks, landscape images (12 per block) were presented for 2.5 seconds. The primary contrast of interest for this study was novel minus familiar trials. We also considered novel minus baseline and familiar minus baseline trials in follow-up analyses.

### MR Image Processing

Image acquisition is described in full in Supplement, section S1. Echo-planar imaging time-series preprocessing (83) used a combination of fMRIPrep (version 20.1.1) (84,85) and XCP engine (86) pipelines (version 1.2.1). First, T1-weighted images were corrected for b0 inhomogeneities (N4BiasFieldCorrection), skull stripped (antsBrainExtraction), classified into tissue type (gray matter, white matter, cerebrospinal fluid; FSL-FAST), put into template space (International Consortium for Brain Mapping 152 nonlinear asymmetrical; antsRegistration), and used as a reference throughout the fMRI preprocessing pipeline. Distortion correction was estimated using ANTS’ symmetrical normalization tool, and FSL-MCFLIRT was used for coregistration. Finally, independent component analysis–based motion artifact removal was performed on the normalized and spatially smoothed (6-mm Gaussian) time series and bandpass filtered (0.01–0.08 Hz Butterworth) for detrending and high-frequency noise removal.

Postprocessing was performed using FSL (http://www.fmrib.ox.ac.uk/fsl) (87). Specifically, the functional data were analyzed implementing General Linear Model in FEAT (version 6.00). Three explanatory variables (EVs) were modeled: novel, familiar, and baseline. Each EV was boxcar convolved with a double-gamma hemodynamic response function for regression. Parameter estimates (*z* scores) for the contrasts of interest (primary outcome: novel vs. familiar; sensitivity analyses: novel vs. baseline and familiar vs. baseline) were derived on a voxelwise basis.

### MR Image Feature Extraction

An a priori hypothesis–based analytical approach was used to focus on limbic activation while improving sensitivity and limiting false positive detection. Regions of interest (ROIs) were limited to the amygdala and hippocampus. We divided the hippocampal ROI into posterior (body and tail) and anterior (head) for both empirical and conceptual considerations. Empirically, head and tail activation are not well correlated. Conceptually, the spatial heterogeneity of hippocampal regions is recognized as important in episodic memory versus emotional memory. ROIs were defined based on the Harvard-Oxford atlas (88) and used to extract regional means for further analysis.

A secondary outcome was derived based on sex-specific findings in the primary analysis suggesting that ELA is related to the amygdala response to novel stimuli. Specifically, PPI parameter estimates were derived that are reflective of the degree to which the right (R) amygdala integrates (signal correlation) with the other ROIs based on task condition. This model was specified in FSL’s FEAT using main effects of task condition and right amygdala signal and the interaction of the two (i.e., signal correlation conditioned on task condition). Collectively, the above analyses were designed to capture 2 different but related constructs of function: 1) traditional amygdala/hippocampal response to task conditions and 2) task condition–dependent integration/correlation with the right amygdala.

### Statistical Modeling Approach

Preliminary analyses were conducted using parameter estimates to characterize baseline activation (novel vs. familiar) and its association with age and sex. Three independent mixed-effects regression models were developed, each for a different ROI. The *p* values reported throughout are uncorrected for multiple comparisons due to the relatively small number of comparisons (three) in the primary hypothesis-based analyses and/or the secondary nature of follow-up analyses (potential confounding covariates and psychophysiological interaction). The models were formulated to predict the bilateral average of each ROI as a function of age and sex, with random intercepts for participants to account for intraparticipant variability. Effect sizes (beta values) are reported in standard deviation units.

Primary analyses considered sex-specific activation (novel vs. familiar) in the context of early-life unpredictability (QUIC). Parsimonious mixed-effects models (main effects of QUIC scores, age, and sex, with random intercepts for participants) were developed to identify the main effect of QUIC scores on activation (novel vs. familiar parameter estimates derived at the visit level in FSL as described above) and followed by repeating this model with an additional QUIC × sex interaction term. Sex-specific slopes were calculated to further interpret the interaction terms based on the full model–estimated beta coefficients and standard errors. While these analyses provided initial insights, additional sensitivity analyses were conducted to test their robustness rigorously and extend the findings. Specifically, we considered 1) lateralized effects, 2) sensitivity to how age was modeled (oldest visit only, aggregating across visits, and limiting the age range [10–13 years]), and 3) potentially confounding factors (negative life events [NLEs] and income-to-needs ratio [INR]). A total of 150 individuals with a total of 223 visits were available for primary analysis that included QUIC measurement. For a consideration of ages used in the current study, see Figure 1 for a distribution of age at visit and the Supplement for an in-depth consideration of the age range in the context of the current study’s findings (see Supplement, section S3). Mixed models were conducted using MATLAB (version R2023b; The MathWorks, Inc.) and its *fitlme* function.

Secondary analyses using PPI measures were conducted to further extend the findings with respect to condition-dependent integration with the right amygdala. These analyses were conducted in 2 stages. The first stage constitutes PPI feature extraction and is described above (see MR Image Feature Extraction). The second stage then repeats the mixed models used above (i.e., main effects of QUIC, age, sex, and QUIC × sex interaction) with the PPI parameter estimate as the outcome. This analysis results in a total of 5 models. Specifically, we tested for sex-specific associations in the condition-dependent connectivity between the right amygdala and the remaining 5 lateral ROIs (left [L] amygdala, left/right head of the hippocampus [headHipp], left/right tail of the hippocampus).

### Covariates

Sex was determined based on biological assignment at birth, and gender was self-reported. It should be noted that 1 participant reported a gender different from their assignment at birth. This individual was considered in a sensitivity analysis to determine the effect of using self-reported gender rather than biological assignment at birth. The analysis showed minimal effect, so we report results only for analyses using biological assignment at birth. INR and childhood experiences of NLEs were assessed via self-report completed at the time of QUIC assessment. INR was computed applying federal guidelines based on number of individuals in the household and annual household income. Participants completed the Coddington Life Events scale, which assesses major life events including death of a parent, parental divorce, jail sentence of a parent, or serious illness requiring hospitalization. INR and life events total score were used as covariates to test the association with unpredictability beyond these more established adversities (89). The multiple imputation package in R was used to account for a total of 5 missing INR values (“mice,” 20 imputations, predictive mean matching).

## Results

### Novel Versus Familiar BOLD Contrast Is Independent of Sex and Age at MRI

Significant group activation (novel vs. familiar contrast) was observed in the hippocampal head ($\hat{\beta }$ = −0.26, *t*_238_ = −3.7, *p* < .001) but not the tail of the hippocampus ($\hat{\beta }$ = 0.01, *t*_238_ = .10, *p* = .92) or amygdala ($\hat{\beta }$ = −0.10, *t*_238_ = −1.5, *p* = .14). The direction of effect suggests greater activation in the head of the hippocampus when presented with familiar relative to novel stimuli. The novel relative to familiar BOLD contrast was not associated with age, sex, or age in a sex-specific manner (i.e., age × sex interaction) in any of the 3 bilateral ROIs. Both novel relative to baseline ($\hat{\beta }$_amyg,group,novel_ = 0.66, *t*_238,__amyg,group,novel_ = 6.3, *p*_amyg,group,novel_ < 10^−8^) and familiar relative to baseline conditions ($\hat{\beta }$_amyg,group,novel_ = 0.67, *t*_238,__amyg,group,novel_ = 7.7, *p*_amyg,group,novel_ < 10^−8^) elicited significant group-level task activation in the amygdala.

### Hippocampus and Amygdala Novelty Signals Were Associated With Exposure to Early-Life Unpredictability in a Sex-Specific Manner

**Figure 3:**
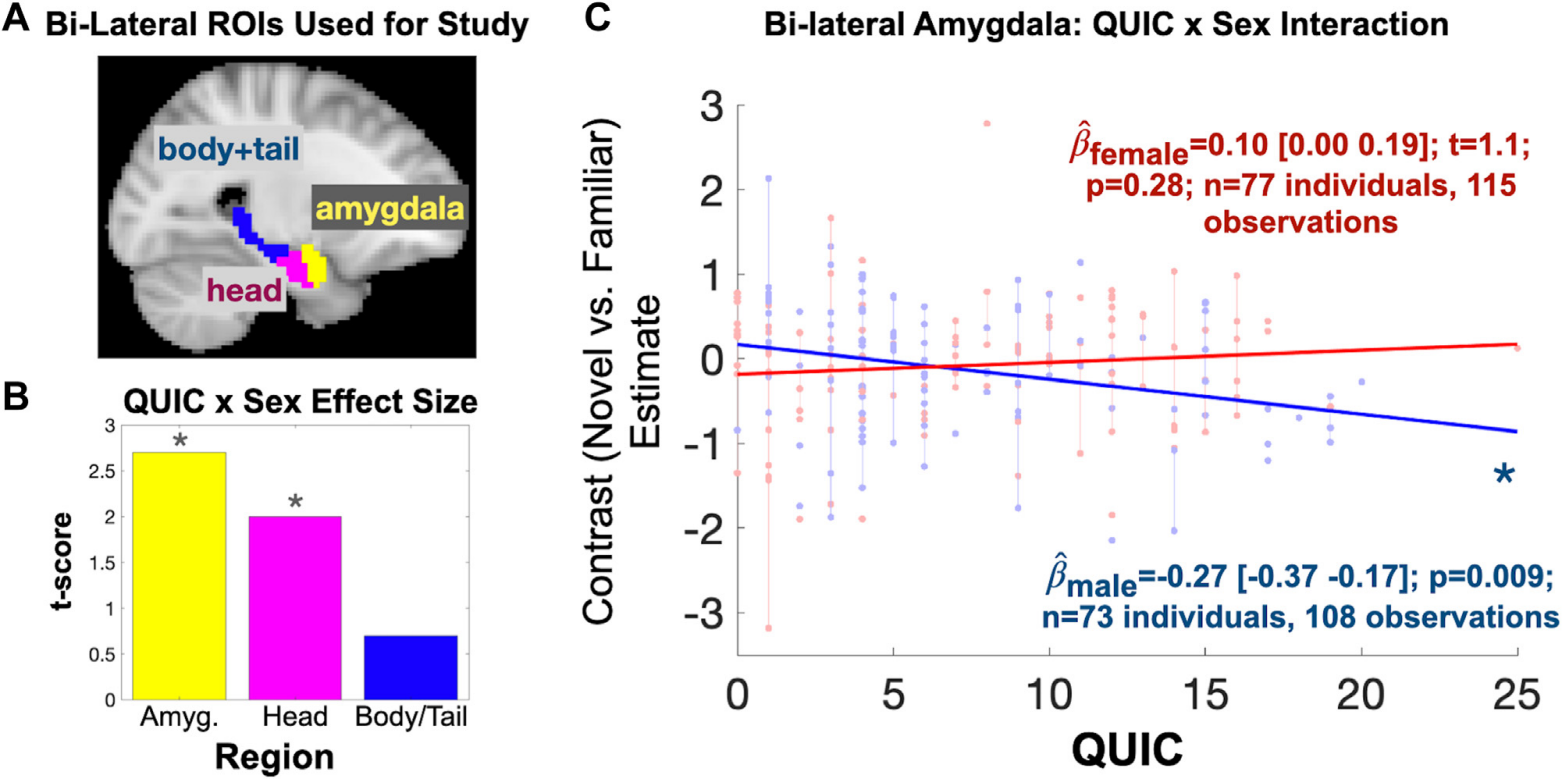
Early-life unpredictability (Questionnaire of Unpredictability in Childhood [QUIC]) is associated with amygdala and hippocampal head activation to novel vs. familiar scenes in a sex-specific manner. **(A)** The amygdala and hippocampus (split by head and body/tail) were considered for analysis. **(B)** The amygdala and head of the hippocampus functional blood oxygen level–dependent activation were associated with QUIC scores in a sex-specific manner (∗*p* < .05). **(C)** Scatter plot depicting sex-specific associations. Red and blue lines give fitted values for girls and boys, respectively. Amyg., amygdala; ROI, region of interest.

No main effect of QUIC scores was observed on the novel relative to familiar BOLD contrast in a parsimonious model that did not include the interaction term. Notably, there was a significant QUIC score × sex interaction (Figure 3) in the head of the hippocampus ($\hat{\beta }$ = 0.14, *t*_218_ = 2.0, *p* = .047) and amygdala ($\hat{\beta }$ = 0.19, *t*_218_ = 2.7, *p* = .007). Specifically, the novel versus familiar BOLD contrast was negatively associated with QUIC scores in males ($\hat{\beta }$_headHipp,M_ = −0.20, *t*_218,__headHipp,M_ = −2.1, *p*_headHipp,M_ = .040; $\hat{\beta }$_amyg,M_ = −0.27, *t*_218,__amyg,M_ = −2.7, *p*_amyg,M_ = .009). In contrast, the effect was not present in females ($\hat{\beta }$_headHipp,F_ = 0.06, *t*_218,__headHipp,F_ = 0.7, *p*_headHipp,F_ = .49; $\hat{\beta }$_amyg,F_ = 0.10, *t*_218,__amyg,F_ = 1.1, *p*_amyg,F_ = .28). Additional analyses suggested that these associations were specific to unpredictability: They largely persist over and above potentially confounding factors that reflect traditional measures (NLEs and INR) of ELA, with the exception of the head of the hippocampus, which showed a marginally significant effect (confound-adjusted QUIC by sex statistics without imputation: *n* = 147; $\hat{\beta }$_amyg_ = 0.19, *t*_210,__amyg_ = 2.6, *p*_amyg_ = .009; $\hat{\beta }$_headHipp_ = 0.12, *t*_210,__headHipp_ = 1.9, *p*_headHipp_ = .062) (pooled confidence intervals with imputation: *N* = 150; $\hat{\beta }$_amyg_ = 0.15 ± 0.07 [SE]; 95% CI, 0.04 to 0.32; $\hat{\beta }$_headHipp_ = 0.12 ± 0.06 [SE]; 95% CI, −0.01 to 0.26). Notably, a diagnostic analysis of variance inflation factors (VIFs) did not present multicollinearity concerns (VIF_unpredictability_ = 1.22, VIF_INR_ = 1.05, and VIF_NLE_ = 1.23). Sensitivity analyses provided 2 additional observations: 1) a modest left-right asymmetry (see Supplement, section S2) was quantitatively and qualitatively observed, with the largest effect (by magnitude and volume) in the right amygdala, which partly motivated our focusing on the right amygdala for secondary PPI analyses; and 2) sex-specific amygdala associations were robust to modeling and sample selection with respect to age at visit (see Supplement, section S3). Collectively, these findings provide robust evidence that early-life unpredictability is negatively associated with the neural response of key limbic structures to novel scenes in a sex-specific manner.

### Amygdala-Hippocampal Connectivity Is Associated With Early-Life Unpredictability in a Sex- and Condition-Specific Manner

**Figure 4:**
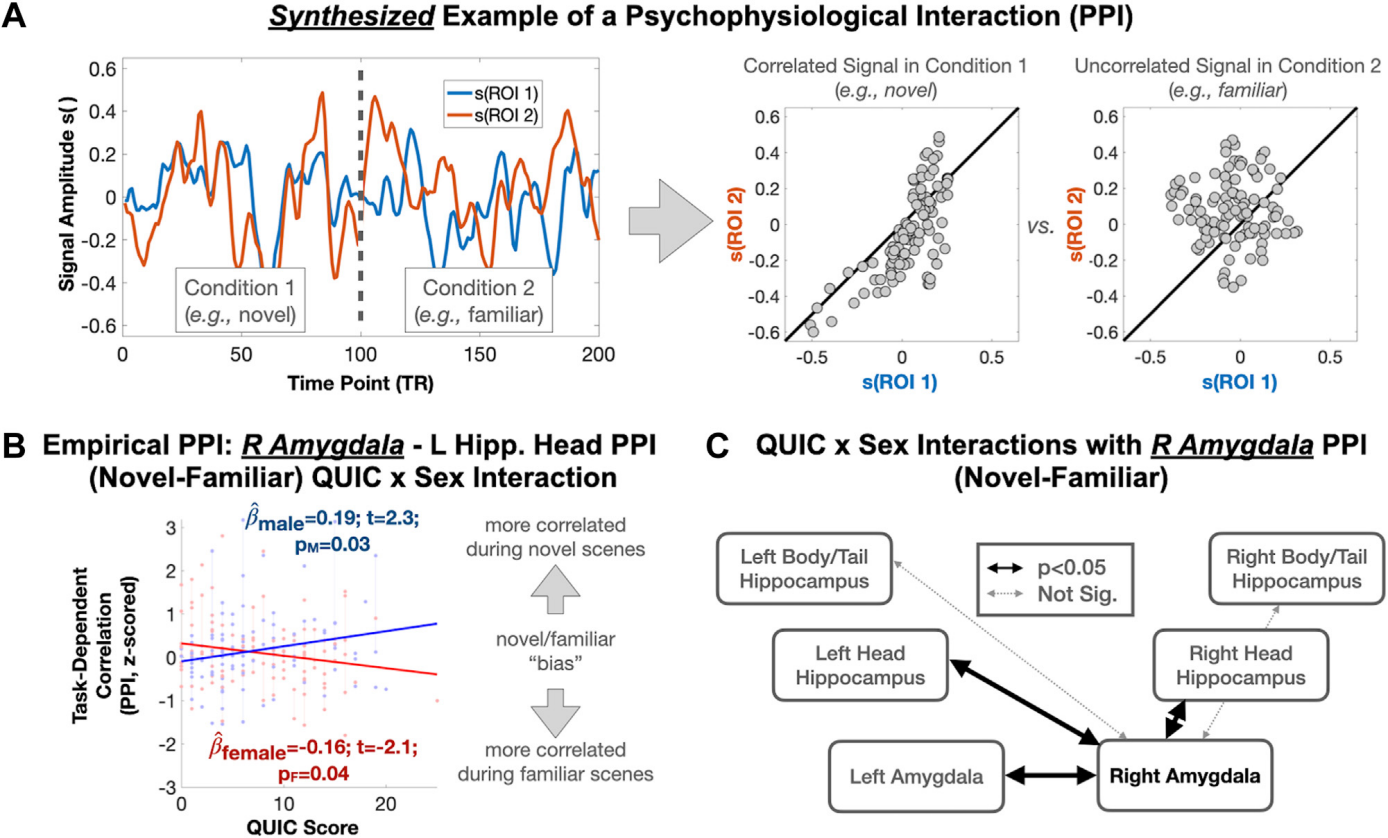
Early-life unpredictability is associated with task-based functional connectivity of the right (R) amygdala in a sex-specific manner. **(A)** Left: simulated examples of raw signals occurring for 1 individual in a PPI. Right: task-dependent correlation structure obtained by correlating signals across regions of interest (ROIs) for each condition. **(B)** Empirical scatter plot showing sex-specific associations between early-life unpredictability (Questionnaire of Unpredictability in Childhood [QUIC]) and PPI-based task-dependent correlations. **(C)** QUIC score × sex interactions are depicted across the observed amygdala and hippocampus (Hipp.) ROIs. L, left; sig., significant.

Based on the above findings, an analysis was performed to probe the sex-specific associations between QUIC scores and PPI measures (i.e., right amygdala connectivity to its lateral analog and the left/right head/tail of the hippocampus in a condition-specific manner). No main effects of QUIC on PPI measures were observed in a parsimonious model that did not include the interaction term. A significant QUIC score × sex interaction was observed in the context of condition-specific connectivity between the right amygdala and the left amygdala (*t*_218,__PPI,RAmyg,LAmyg_ = −2.9, *p*_PPI,RAmyg,LAmyg_ = .004), as well as between the right amygdala and the left/right head, but not the tail, of the hippocampus (*t*_218,__PPI,RAmyg,LheadHipp_ = −3.1, *p*_PPI,RAmyg,LheadHipp_ = .003; *t*_218,__PPI,RAmyg,RheadHipp_ = −2.1, *p*_PPI,RAmyg,RheadHipp_ = .040) (Figure 4). While we present and interpret unadjusted *p* values here due to the limited number of comparisons (five) and secondary nature of the analysis, we note that 2 of the 3 observed findings survived Bonferroni correction at a significance threshold of .01 (i.e., .05/5). These sex-specific connectivity analyses provide evidence that the association between QUIC scores and condition-specific connectivity with the right amygdala was largely significant in both boys and girls, but in opposing directions (*t*_218,__PPI,RAmyg,LAmyg,M_ = 1.6, *p*_PPI,RAmyg,LAmyg,M_ = .10; *t*_218,__PPI,RAmyg,LAmyg,F_ = −2.5, *p*_PPI,RAmyg,LAmyg,F_ = .014; *t*_218,__PPI,RAmyg,LheadHipp,M_ = 2.3, *p*_PPI,RAmyg,LheadHipp,M_ = .025; *t*_218,__PPI,RAmyg,LheadHipp,F_ = −2.1, *p*_PPI,RAmyg,LheadHipp,F_ = .039). Broadly, these findings suggest that functional connectivity to the amygdala in males exposed to early childhood unpredictability is relatively greater in the novel condition, whereas in females, functional connectivity to the amygdala is relatively greater in the familiar condition. Thus, this finding supports the idea that early-life unpredictability is associated with sex-specific patterns of limbic connectivity.

## Discussion

This study provides new evidence that exposure to early-life unpredictability has sex-specific implications for the functional organization of limbic circuits in the context of their responses to novelty. Specifically, we observed sex-specific associations between early-life unpredictability and limbic regional (hippocampus and amygdala) BOLD activation/connectivity in response to novel relative to familiar scenes. In boys, we observed that higher early-life unpredictability was associated with decreased limbic BOLD activation in response to novel stimuli. In girls, higher unpredictability was associated with decreased condition-specific (novel relative to familiar) functional connectivity to the amygdala, while the opposite pattern was observed in boys. Taken together, these findings suggest that early-life unpredictability has ramifications for both male and female limbic system development in the context of their responses to novelty but manifests in different ways. Importantly, the observed associations were not explained by more commonly studied forms of ELA such as socioeconomic disadvantage and NLEs. This work adds to a growing body of evidence suggesting that the effects of early-life unpredictability on neurodevelopment may be distinct from those reflective of overall exposure to adversity.

In this study, we utilized a hypothesis-driven approach designed to understand the effects of exposure to early-life unpredictability on functional activation and connectivity within and between key brain regions involved in learning novel experiences, specifically the amygdala and hippocampus. We posited that exposure to unpredictable signals from the parents and environment during a sensitive developmental period may alter processing of neural responses to novelty later in life. Consistent with this concept, we found that the amygdala and hippocampus are functionally altered in response to early-life unpredictability.

The amygdala has a well-established role in emotional processing (90) and stress during development (41), motivating its widespread use as a target for understanding the putative pathway between ELA and mental health outcomes (91, 92, 93, 94). Notably, the modest amygdala asymmetry observed in our study is consistent with previous findings (95). Our sex-specific findings in the context of unpredictability join a diverse body of work demonstrating amygdala activation and connectivity changes in response to various forms of ELA, including maltreatment (56) and threat (96). Importantly, our observations suggest that unpredictability may uniquely shape amygdala function independent of other established forms of ELA, such as exposure to NLEs and socioeconomic status.

In the hippocampus, we observed a significant functional distinction between the head (anterior) and the body/tail (posterior) regions, particularly in response to novel stimuli. The head also showed more pronounced condition-specific integration with the amygdala, a key center for emotional processing. These findings are consistent with the anterior-posterior emotion-cognition segregation hypothesis (97, 98, 99), which posits that the head of the hippocampus is differentially involved in emotional processing relative to the body/tail region. While this anterior-posterior functional gradation is more pronounced in rodent models and remains debated in humans, our findings of increased gradation in individuals exposed to unpredictable early-life environments suggest that this gradient may become more distinct following developmental insults.

We report significant and opposing effects of unpredictability in boys and girls, highlighting sex differences in the processing of novel stimuli. Our observations suggest that early-life unpredictability may either be encoded differently in males and females or may be similarly encoded but lead to differences in outcomes by sex. Our data in experimental animals suggest that distinct, partially overlapping neuronal populations encode early-life unpredictability in males and females (71), and unpredictability exerts sex-specific enduring influences on the fine innervation patterns in limbic circuits (73). These circuit alterations may lead to a neural response bias toward novel or familiar stimuli, with this bias differing between males and females in response to ELA. These findings may have implications for how individuals process information in novel (e.g., first day of school) versus familiar (e.g., end of the school year) situations, with sex-specific impacts of ELA potentially contributing to differences in mental health disorders (e.g., anxiety and/or withdrawn behaviors). Notably, these concepts are consistent with an emerging body of preclinical evidence illustrating distinct phenotypic consequences of early-life unpredictability in male and female rodents that emerge in early life (71). For example, early-life unpredictability has been experimentally shown to increase anhedonic behavior in males, while unpredictable signals have been linked to increases in hedonic (e.g., drug-seeking) behaviors in females (38,72,100,101). Consistent with these observations, the current findings highlight the importance of considering sex differences in the study of ELA and its long-term effects on mental health (24,75).

The current study’s limitations should be carefully considered. First, we did not observe a significant novel-familiar contrast in the amygdala at the group level. This observation is consistent with previous literature suggesting that human representation (e.g., faces) is necessary for eliciting group-level activation in the amygdala (102) and limits the interpretability of amygdala responses as an index of novelty processing in this context. Next, we did not include behavioral outcomes such as novelty seeking, internalizing, or risk-taking behaviors. Although we contextualize our findings within the existing literature, further research is necessary to directly link the observed neural patterns with their behavioral relevance and potential mediating roles. Meta-analyses suggest that adversity-related differences in amygdala function are observed in adults only (103) and that while early adversity effects converge on amygdala (and putamen) function, they appear to be specific to emotion tasks (104). While our study focuses on sex-specific associations rather than group-level activation differences, these studies highlight the importance of developmental stage and task specificity when interpreting amygdala responses to novelty. Future studies might consider sex-specific study designs that extend to emotion and reward-based paradigms across development. Additionally, condition-specific approaches to characterizing functional connectivity have a demonstrated potential to induce spurious findings as they rely on precise specification of task-based activation for nuisance regression (105). While we cannot entirely rule this out, the choice of block-designed stimuli minimizes misspecification errors associated with onset and cessation of stimuli, and furthermore, the associations with unpredictable early-life experiences are unlikely to be confounded in this case. It should also be noted that the findings limited here to the hippocampus and amygdala are in the context of a task designed to elicit responses to novelty. Given the broader extent of the brain’s response to novelty, the observed effects may be downstream or in addition to those of other brain structures in this broader network. Unpredictability was assessed with the QUIC, a validated self-report measure of childhood experiences of unpredictability. As with any self-report measure, there is a possibility of reporting bias. The QUIC has been validated with prospective observational measures of unpredictability as well as with prospective measures of experience of items assessed on the QUIC (45). Furthermore, participants were assessed during adolescence, and future work should evaluate long-term consequences of exposure to childhood unpredictability with the inclusion of adult samples. Finally, while the current sample and study design may be sufficiently powered, the typically small magnitude of brain-behavior effects and novelty of findings suggests the need for future external validation studies. Notably, the upcoming HBCD (HEALthy Brain and Child Development) study, which will include the QUIC (46) concurrent with early-life MRI assessments from birth to age 6 years (106), provides an opportunity to generalize the current work and to explore the timing and onset of effects, with the inclusion of prospective assessments of unpredictability and MRI in early life.

### Conclusions

Our findings demonstrate that unpredictability in early life differentially impacts the functional activation and connectivity of key limbic regions—the amygdala and hippocampus—in the context of novel versus familiar stimuli and that these effects are sex dependent. Importantly, we examine a novel form of adversity (unpredictable signals), which exerts enduring consequences beyond established types of ELA, such as exposure to NLEs and income. By identifying unpredictable signals as a distinct form of adversity with lasting neurodevelopmental consequences, our study further highlights the importance of stable early environments (100,107).

## Supplementary Material

Supplemental Methods, Results, Figures S1–S3, and Tables S1–S2

Key Resources Table

## References

[1] Sheridan M.A., McLaughlin K.A. (2014). Dimensions of early experience and neural development: Deprivation and threat. Trends Cogn Sci.

[2] Smith K.E., Pollak S.D. (2021). Rethinking concepts and categories for understanding the neurodevelopmental effects of childhood adversity. Perspect Psychol Sci.

[3] Luby J.L., Barch D., Whalen D., Tillman R., Belden A. (2017). Association between early life adversity and risk for poor emotional and physical health in adolescence: A putative mechanistic neurodevelopmental pathway. JAMA Pediatr.

[4] Nelson C.A., Bhutta Z.A., Burke Harris N., Danese A., Samara M. (2020). Adversity in childhood is linked to mental and physical health throughout life. BMJ.

[5] Machlin L., Egger H.L., Stein C.R., Navarro E., Carpenter K.L.H., Goel S. (2023). Distinct associations of deprivation and threat with alterations in brain structure in early childhood. J Am Acad Child Adolesc Psychiatry.

[6] Murgueitio N., Sheridan M.A., Shipkova M., Halberstadt A.G., Garrett-Peters P.T., Propper C.B. (2025). Developmental impacts of deprivation and threat on emotion recognition. Neurotoxicol Teratol.

[7] Narayan A.J., Merrick J.S., Lane A.S., Larson M.D. (2023). A multisystem, dimensional interplay of assets versus adversities: Revised benevolent childhood experiences (BCEs) in the context of childhood maltreatment, threat, and deprivation. Dev Psychopathol.

[8] Penner F., Khoury J.E., Bosquet Enlow M., Lyons-Ruth K. (2023). Threat versus deprivation in mother’s childhood: Differential relations to hair cortisol and psychopathology in pregnancy. Child Abuse Negl.

[9] Sadikova E., Weissman D.G., Rosen M.L., Robinson E., Lengua L.J., Sheridan M.A. (2025). Identifying cognitive, affective, and developmental mechanisms linking threat and deprivation with adolescent psychopathology. J Child Psychol Psychiatry.

[10] Wang X., Lu J., Liu Q., Yu Q., Fan J., Gao F. (2022). Childhood experiences of threat and deprivation predict distinct depressive symptoms: A parallel latent growth curve model. J Affect Disord.

[11] Callaghan B.L., Gee D.G., Gabard-Durnam L., Telzer E.H., Humphreys K.L., Goff B. (2019). Decreased amygdala reactivity to parent cues protects against anxiety following early adversity: An examination across 3 years. Biol Psychiatry Cogn Neurosci Neuroimaging.

[12] Gao W., Grewen K., Knickmeyer R.C., Qiu A., Salzwedel A., Lin W., Gilmore J.H. (2019). A review on neuroimaging studies of genetic and environmental influences on early brain development. Neuroimage.

[13] Lambert H.K., Peverill M., Sambrook K.A., Rosen M.L., Sheridan M.A., McLaughlin K.A. (2019). Altered development of hippocampus-dependent associative learning following early-life adversity. Dev Cogn Neurosci.

[14] Lambert H.K., Sheridan M.A., Sambrook K.A., Rosen M.L., Askren M.K., McLaughlin K.A. (2017). Hippocampal contribution to context encoding across development is disrupted following early-life adversity. J Neurosci.

[15] Sheridan M.A., Peverill M., Finn A.S., McLaughlin K.A. (2017). Dimensions of childhood adversity have distinct associations with neural systems underlying executive functioning. Dev Psychopathol.

[16] Surani Z., Turesky T.K., Sullivan E., Shama T., Haque R., Islam N. (2025). Examining the relationship between psychosocial adversity and inhibitory control: A functional magnetic resonance imaging study of children growing up in extreme poverty. J Exp Child Psychol.

[17] Vannucci A., Fields A., Hansen E., Katz A., Kerwin J., Tachida A. (2023). Interpersonal early adversity demonstrates dissimilarity from early socioeconomic disadvantage in the course of human brain development: A meta-analysis. Neurosci Biobehav Rev.

[18] VanTieghem M., Korom M., Flannery J., Choy T., Caldera C., Humphreys K.L. (2021). Longitudinal changes in amygdala, hippocampus and cortisol development following early caregiving adversity. Dev Cogn Neurosci.

[19] Xia C.H., Ma Z., Ciric R., Gu S., Betzel R.F., Kaczkurkin A.N. (2018). Linked dimensions of psychopathology and connectivity in functional brain networks. Nat Commun.

[20] Ingalhalikar M., Smith A., Parker D., Satterthwaite T.D., Elliott M.A., Ruparel K. (2014). Sex differences in the structural connectome of the human brain. Proc Natl Acad Sci U S A.

[21] Baldwin J.R., Caspi A., Meehan A.J., Ambler A., Arseneault L., Fisher H.L. (2021). Population vs individual prediction of poor health from results of adverse childhood experiences screening. JAMA Pediatr.

[22] Glynn L.M., Howland M.A., Sandman C.A., Davis E.P., Phelan M., Baram T.Z., Stern H.S. (2018). Prenatal maternal mood patterns predict child temperament and adolescent mental health. J Affect Disord.

[23] Noroña-Zhou A.N., Morgan A., Glynn L.M., Sandman C.A., Baram T.Z., Stern H.S., Davis E.P. (2020). Unpredictable maternal behavior is associated with a blunted infant cortisol response. Dev Psychobiol.

[24] Spadoni A.D., Vinograd M., Cuccurazzu B., Torres K., Glynn L.M., Davis E.P. (2022). Contribution of early-life unpredictability to neuropsychiatric symptom patterns in adulthood. Depress Anxiety.

[25] Davis E.P., Korja R., Karlsson L., Glynn L.M., Sandman C.A., Vegetabile B. (2019). Across continents and demographics, unpredictable maternal signals are associated with children’s cognitive function. EBioMedicine.

[26] Holmberg E., Kataja E.L., Davis E.P., Pajulo M., Nolvi S., Lahtela H. (2022). Unpredictable maternal sensory signals in caregiving behavior are associated with child effortful control. PLoS One.

[27] Ellis B.J., Sheridan M.A., Belsky J., McLaughlin K.A. (2022). Why and how does early adversity influence development? Toward an integrated model of dimensions of environmental experience. Dev Psychopathol.

[28] Forest T.A., McCormick S.A., Davel L., Mlandu N., Zieff M.R., Khula South Africa Data Collection Team (2025). Early caregiver predictability shapes neural indices of statistical learning later in infancy. Dev Sci.

[29] Martinez J.L., Hasty C., Morabito D., Maranges H.M., Schmidt N.B., Maner J.K. (2022). Perceptions of childhood unpredictability, delay discounting, risk-taking, and adult externalizing behaviors: A life-history approach. Dev Psychopathol.

[30] Ugarte E., Hastings P.D. (2023). Assessing unpredictability in caregiver-child relationships: Insights from theoretical and empirical perspectives. Dev Psychopathol.

[31] Furtado E.J., Camacho M.C., Chin J.H., Barch D.M. (2024). Complex emotion processing and early life adversity in the Healthy Brain Network sample. Dev Cogn Neurosci.

[32] Munakata Y., Placido D., Zhuang W. (2023). What’s next? Advances and challenges in understanding how environmental predictability shapes the development of cognitive control. Curr Dir Psychol Sci.

[33] Davis E.P., McCormack K., Arora H., Sharpe D., Short A.K., Bachevalier J. (2022). Early life exposure to unpredictable parental sensory signals shapes cognitive development across three species. Front Behav Neurosci.

[34] Davis E.P., Stout S.A., Molet J., Vegetabile B., Glynn L.M., Sandman C.A. (2017). Exposure to unpredictable maternal sensory signals influences cognitive development across species. Proc Natl Acad Sci U S A.

[35] Molet J., Maras P.M., Kinney-Lang E., Harris N.G., Rashid F., Ivy A.S. (2016). MRI uncovers disrupted hippocampal microstructure that underlies memory impairments after early-life adversity. Hippocampus.

[36] Glynn L.M., Davis E.P., Luby J.L., Baram T.Z., Sandman C.A. (2021). A predictable home environment may protect child mental health during the COVID-19 pandemic. Neurobiol Stress.

[37] Birnie M.T., Short A.K., de Carvalho G.B., Taniguchi L., Gunn B.G., Pham A.L. (2023). Stress-induced plasticity of a CRH/GABA projection disrupts reward behaviors in mice. Nat Commun.

[38] Levis S.C., Baram T.Z., Mahler S.V. (2022). Neurodevelopmental origins of substance use disorders: Evidence from animal models of early-life adversity and addiction. Eur J Neurosci.

[39] Levis S.C., Birnie M.T., Bolton J.L., Perrone C.R., Montesinos J.S., Baram T.Z., Mahler S.V. (2022). Enduring disruption of reward and stress circuit activities by early-life adversity in male rats. Transl Psychiatry.

[40] Xu Y., Harms M.B., Green C.S., Wilson R.C., Pollak S.D. (2023). Childhood unpredictability and the development of exploration. Proc Natl Acad Sci U S A.

[41] Howell B.R., McMurray M.S., Guzman D.B., Nair G., Shi Y., McCormack K.M. (2017). Maternal buffering beyond glucocorticoids: Impact of early life stress on corticolimbic circuits that control infant responses to novelty. Soc Neurosci.

[42] Edmiston E.K., Blackford J.U. (2013). Childhood maltreatment and response to novel face stimuli presented during functional magnetic resonance imaging in adults. Psychiatry Res Neuroimaging.

[43] Aloi J., Crum K.I., Blair K.S., Zhang R., Bashford-Largo J., Bajaj S. (2024). Childhood neglect is associated with alterations in neural prediction error signaling and the response to novelty. Psychol Med.

[44] Aran Ö., Swales D.A., Bailey N.A., Korja R., Holmberg E., Eskola E. (2024). Across ages and places: Unpredictability of maternal sensory signals and child internalizing behaviors. J Affect Disord.

[45] Glynn L.M., Stern H.S., Howland M.A., Risbrough V.B., Baker D.G., Nievergelt C.M. (2019). Measuring novel antecedents of mental illness: The Questionnaire of Unpredictability in Childhood. Neuropsychopharmacology.

[46] Lindert N.G., Maxwell M.Y., Liu S.R., Stern H.S., Baram T.Z., Poggi Davis E. (2022). Exposure to unpredictability and mental health: Validation of the brief version of the Questionnaire of Unpredictability in Childhood (QUIC-5) in English and Spanish. Front Psychol.

[47] Liu S., Bailey N., Romero-González S., Moors A., Campos B., Davis E.P., Glynn L.M. (2025). The QUIC-SP: A Spanish language tool assessing unpredictability in early life is linked to physical and mental health. PLoS One.

[48] Kumaran D., Maguire E.A. (2009). Novelty signals: A window into hippocampal information processing. Trends Cogn Sci.

[49] Blackford J.U., Buckholtz J.W., Avery S.N., Zald D.H. (2010). A unique role for the human amygdala in novelty detection. Neuroimage.

[50] Gee D.G., Gabard-Durnam L.J., Flannery J., Goff B., Humphreys K.L., Telzer E.H. (2013). Early developmental emergence of human amygdala-prefrontal connectivity after maternal deprivation. Proc Natl Acad Sci U S A.

[51] Tottenham N., Hare T.A., Millner A., Gilhooly T., Zevin J.D., Casey B.J. (2011). Elevated amygdala response to faces following early deprivation. Dev Sci.

[52] Demers C.H., Hankin B.L., Hennessey E.M.P., Haase M.H., Bagonis M.M., Kim S.H. (2022). Maternal adverse childhood experiences and infant subcortical brain volume. Neurobiol Stress.

[53] Dong D., Ironside M., Belleau E.L., Sun X., Cheng C., Xiong G. (2022). Sex-specific neural responses to acute psychosocial stress in depression. Transl Psychiatry.

[54] Etkin A., Wager T.D. (2007). Functional neuroimaging of anxiety: A meta-analysis of emotional processing in PTSD, social anxiety disorder, and specific phobia. Am J Psychiatry.

[55] Price R.B., Duman R. (2020). Neuroplasticity in cognitive and psychological mechanisms of depression: An integrative model. Mol Psychiatry.

[56] Tottenham N., Sheridan M.A. (2010). A review of adversity, the amygdala and the hippocampus: A consideration of developmental timing. Front Hum Neurosci.

[57] Ben-Zion Z., Korem N., Fine N.B., Katz S., Siddhanta M., Funaro M.C. (2024). Structural neuroimaging of hippocampus and amygdala subregions in posttraumatic stress disorder: A scoping review. Biol Psychiatry Glob Open Sci.

[58] Forstmeier W., Wagenmakers E.J., Parker T.H. (2017). Detecting and avoiding likely false-positive findings - A practical guide. Biol Rev Camb Philos Soc.

[59] Marek S., Tervo-Clemmens B., Calabro F.J., Montez D.F., Kay B.P., Hatoum A.S. (2022). Reproducible brain-wide association studies require thousands of individuals. Nature.

[60] Hankin B.L. (2009). Development of sex differences in depressive and co-occurring anxious symptoms during adolescence: Descriptive trajectories and potential explanations in a multiwave prospective study. J Clin Child Adolesc Psychol.

[61] Hammerslag L.R., Gulley J.M. (2016). Sex differences in behavior and neural development and their role in adolescent vulnerability to substance use. Behav Brain Res.

[62] McHugh R.K., Votaw V.R., Sugarman D.E., Greenfield S.F. (2018). Sex and gender differences in substance use disorders. Clin Psychol Rev.

[63] Goel N., Bale T.L. (2008). Organizational and activational effects of testosterone on masculinization of female physiological and behavioral stress responses. Endocrinology.

[64] Schulz K.M., Molenda-Figueira H.A., Sisk C.L. (2009). Back to the future: The organizational-activational hypothesis adapted to puberty and adolescence. Horm Behav.

[65] Kaplan H.S., Logeman B.L., Zhang K., Yawitz T.A., Santiago C., Sohail N. (2025). Sensory input, sex and function shape hypothalamic cell type development. Nature.

[66] Kaczkurkin A.N., Raznahan A., Satterthwaite T.D. (2019). Sex differences in the developing brain: Insights from multimodal neuroimaging. Neuropsychopharmacology.

[67] Clifton V.L. (2010). Review: Sex and the human placenta: Mediating differential strategies of fetal growth and survival. Placenta.

[68] Sandman C.A., Glynn L.M., Davis E.P. (2013). Is there a viability-vulnerability tradeoff? Sex differences in fetal programming. J Psychosom Res.

[69] Bath K.G. (2020). Synthesizing views to understand sex differences in response to early life adversity. Trends Neurosci.

[70] Bale T.L., Epperson C.N. (2015). Sex differences and stress across the lifespan. Nat Neurosci.

[71] Kooiker C.L., Chen Y., Birnie M.T., Baram T.Z. (2023). Genetic tagging uncovers a robust, selective activation of the thalamic paraventricular nucleus by adverse experiences early in life. Biol Psychiatry Glob Open Sci.

[72] Levis S.C., Mahler S.V., Baram T.Z. (2021). The developmental origins of opioid use disorder and its comorbidities. Front Hum Neurosci.

[73] Taniguchi L., Goodpaster C.M., de Carvalho G.B., Birnie M.T., Chen Y., Chen L.Y. (2024). Sex-and stress-dependent plasticity of a corticotropin releasing hormone / GABA projection from the basolateral amygdala to nucleus accumbens that mediates reward behaviors. bioRxiv.

[74] Short A.K., Weber R., Kamei N., Wilcox Thai C., Arora H., Mortazavi A. (2024). Individual longitudinal changes in DNA-methylome identify signatures of early-life adversity and correlate with later outcome. Neurobiol Stress.

[75] Davis E.P., Glynn L.M. (2024). Annual research review: The power of predictability - Patterns of signals in early life shape neurodevelopment and mental health trajectories. J Child Psychol Psychiatry.

[76] Granger S.J., Glynn L.M., Sandman C.A., Small S.L., Obenaus A., Keator D.B. (2021). Aberrant maturation of the uncinate fasciculus follows exposure to unpredictable patterns of maternal signals. J Neurosci.

[77] Wheelock M.D., Hect J.L., Hernandez-Andrade E., Hassan S.S., Romero R., Eggebrecht A.T., Thomason M.E. (2019). Sex differences in functional connectivity during fetal brain development. Dev Cogn Neurosci.

[78] Pedersen W.S., Balderston N.L., Miskovich T.A., Belleau E.L., Helmstetter F.J., Larson C.L. (2017). The effects of stimulus novelty and negativity on BOLD activity in the amygdala, hippocampus, and bed nucleus of the stria terminalis. Soc Cogn Affect Neurosci.

[79] Stern C.E., Corkin S., González R.G., Guimaraes A.R., Baker J.R., Jennings P.J. (1996). The hippocampal formation participates in novel picture encoding: Evidence from functional magnetic resonance imaging. Proc Natl Acad Sci U S A.

[80] Yassa M.A., Stark C.E.L. (2008). Multiple signals of recognition memory in the medial temporal lobe. Hippocampus.

[81] Riley J.D., Chen E.E., Winsell J., Davis E.P., Glynn L.M., Baram T.Z. (2018). Network specialization during adolescence: Hippocampal effective connectivity in boys and girls. Neuroimage.

[82] Hunt C., Vinograd M., Glynn L.M., Davis E.P., Baram T.Z., Stern H.S. (2024). Childhood unpredictability is associated with increased risk for long- and short-term depression and anhedonia symptoms following combat deployment. J Mood Anxiety Disord.

[83] Jirsaraie R.J., Palma A.M., Small S.L., Sandman C.A., Davis E.P., Baram T.Z. (2024). Prenatal exposure to maternal mood entropy is associated with a weakened and inflexible salience network in adolescence. Biol Psychiatry Cogn Neurosci Neuroimaging.

[84] Esteban O., Ciric R., Finc K., Blair R.W., Markiewicz C.J., Moodie C.A. (2020). Analysis of task-based functional MRI data preprocessed with fMRIPrep. Nat Protoc.

[85] Esteban O., Markiewicz C.J., Blair R.W., Moodie C.A., Isik A.I., Erramuzpe A. (2019). fMRIPrep: A robust preprocessing pipeline for functional MRI. Nat Methods.

[86] Mehta K., Salo T., Madison T.J., Adebimpe A., Bassett D.S., Bertolero M. (2024). XCP-D: A Robust Pipeline for the post-processing of fMRI data. Imaging Neuroscience.

[87] Smith S.M., Jenkinson M., Woolrich M.W., Beckmann C.F., Behrens T.E.J., Johansen-Berg H. (2004). Advances in functional and structural MR image analysis and implementation as FSL. Neuroimage.

[88] Frazier J.A., Chiu S., Breeze J.L., Makris N., Lange N., Kennedy D.N. (2005). Structural brain magnetic resonance imaging of limbic and thalamic volumes in pediatric bipolar disorder. Am J Psychiatry.

[89] Coddington R.D. (1972). The Significance of life events as etiologic factors in the diseases of children. II. A study of a normal population. J Psychosom Res.

[90] Davis M., Whalen P.J. (2001). The amygdala: Vigilance and emotion. Mol Psychiatry.

[91] Hanson J.L., Nacewicz B.M. (2021). Amygdala allostasis and early life adversity: Considering excitotoxicity and Inescapability in the sequelae of stress. Front Hum Neurosci.

[92] Sicorello M., Thome J., Herzog J., Schmahl C. (2021). Differential effects of early adversity and posttraumatic stress disorder on amygdala reactivity: The role of developmental timing. Biol Psychiatry Cogn Neurosci Neuroimaging.

[93] Shackman A.J., Grogans S.E., Fox A.S. (2024). Fear, anxiety and the functional architecture of the human central extended amygdala. Nat Rev Neurosci.

[94] Filippi C.A., Ravi S., Bracy M., Winkler A., Sylvester C.M., Pine D.S., Fox N.A. (2021). Amygdala functional connectivity and negative reactive temperament at Age 4 months. J Am Acad Child Adolesc Psychiatry.

[95] Fowler C.H., Bogdan R., Gaffrey M.S. (2021). Stress-induced cortisol response is associated with right amygdala volume in early childhood. Neurobiol Stress.

[96] McLaughlin K.A., Weissman D., Bitrán D. (2019). Childhood adversity and neural development: A systematic review. Annu Rev Dev Psychol.

[97] Fanselow M.S., Dong H.W. (2010). Are the dorsal and ventral hippocampus functionally distinct structures?. Neuron.

[98] Plachti A., Eickhoff S.B., Hoffstaedter F., Patil K.R., Laird A.R., Fox P.T. (2019). Multimodal parcellations and extensive behavioral profiling tackling the hippocampus gradient. Cereb Cortex.

[99] Vos de Wael R., Larivière S., Caldairou B., Hong S.J., Margulies D.S., Jefferies E. (2018). Anatomical and microstructural determinants of hippocampal subfield functional connectome embedding. Proc Natl Acad Sci U S A.

[100] Birnie M.T., Baram T.Z. (2022). Principles of emotional brain circuit maturation. Science.

[101] Kangas B.D., Ang Y.S., Short A.K., Baram T.Z., Pizzagalli D.A. (2024). Computational modeling differentiates learning rate from reward sensitivity deficits produced by early-life adversity in a rodent touchscreen probabilistic reward task. Biol Psychiatry Glob Open Sci.

[102] Balderston N.L., Schultz D.H., Helmstetter F.J. (2011). The human amygdala plays a stimulus specific role in the detection of novelty. Neuroimage.

[103] Hosseini-Kamkar N., Varvani Farahani M., Nikolic M., Stewart K., Goldsmith S., Soltaninejad M. (2023). Adverse life experiences and brain function: A meta-analysis of functional magnetic resonance imaging findings. JAMA Netw Open.

[104] Kraaijenvanger E.J., Pollok T.M., Monninger M., Kaiser A., Brandeis D., Banaschewski T., Holz N.E. (2020). Impact of early life adversities on human brain functioning: A coordinate-based meta-analysis. Neurosci Biobehav Rev.

[105] O’Reilly J.X., Woolrich M.W., Behrens T.E.J., Smith S.M., Johansen-Berg H. (2012). Tools of the trade: Psychophysiological interactions and functional connectivity. Soc Cogn Affect Neurosci.

[106] Edwards R.C., Planalp E.M., Bosquet Enlow M., Akshoomoff N., Bodison S.C., Brennan M.B. (2024). Capturing the complexity of child behavior and caregiver-child interactions in the HEALthy Brain and Child Development (HBCD) Study using a rigorous and equitable approach. Dev Cogn Neurosci.

[107] Glynn L.M., Liu S.R., Lucas C.T., Davis E.P. (2024). Leveraging the science of early life predictability to inform policies promoting child health. Dev Cogn Neurosci.

