# Supplementary material for: Sex-Specific Effects of Early-Life Unpredictability on Hippocampal and Amygdala Responses to Novelty in Adolescents
Source: Biol Psychiatry Glob Open Sci. 2025 Jul 7;5(6):100561. doi: 10.1016/j.bpsgos.2025.100561 (PMC12673399; doi:10.1016/j.bpsgos.2025.100561)
Supplement: Supplemental Methods, Results, Figures S1–S3, and Tables S1–S2 [file mmc1.pdf]

SUPPLEMENTARY INFORMATION

Sex-Specific Effects of Early-Life Unpredictability on Hippocampal and Amygdala Responses to Novelty in Adolescents

Davis *et al.*

Supplement S1: Methods Supplement

Measurement of Early life Unpredictability

Early life unpredictability was characterized using the validated 38-item version of the Questionnaire of Unpredictability in Childhood (QUIC) (1). Example items include, "I experienced changes in my custody arrangement", and "At least one of my parents regularly checked that I did my homework". Notably, the QUIC shows strong internal consistency (α=0.89) and test-retest reliability (r=0.92) (1). The QUIC also demonstrates convergent validity with prospective, observational measures of unpredictability in infancy, such as unpredictable maternal sensory signals (r=0.23) and unpredictability of maternal mood (r =0.17), as well as predictive validity for risk of depression (r=0.42), anhedonia (r=0.37), and anxiety (r=0.43) (1-3)

MR Image Acquisition

Whole-brain MRI was performed using a Philips 3T MRI scanner equipped with a 32-channel head coil. Participants were instructed to remain awake throughout the imaging protocol. T1-weighted 3D MPRAGE (SENSE factor=2.4, 1mm iso, TR/TE=8/3.7 ms, matrix=256×204, 208 slices, FA=8°, scan duration of 9m 46s) images were acquired for anatomical reference. BOLD-weighted images were acquired using a single shot interleaved EPI sequence (flip angle=64°, TR=2.5s, ~3mm iso, TE=30ms, matrix=64x61, 51 slices, SENSE reduction factor of 2.5, scan duration of 6m 40s).

Supplement S2: Laterality Analysis for Investigating Condition-Specific Signal Correlations

In the main manuscript, bilateral ROIs were considered to reduce comparisons and increase sensitivity. However, based on a strong body of literature demonstrating laterality of developmental programming effects in limbic region outcomes (4), we conducted follow-up sensitivity analyses by splitting ROIs into their lateral components. Accordingly, we tested six independent linear mixed effects models (N=150 unique individuals, 223 observations) consistent with those used in the main manuscript. Although the effect sizes (t-score) in the right hemisphere were quantitatively larger for all three regions (Supplementary Table 1), we lacked sufficient power to demonstrate statistically significant hemispheric asymmetry when adjusting for the contralateral hemisphere ($p_{RheadHipp,adjLheadHipp}$=0.089; $p_{RAmyg,adjLAmyg}$=0.152).

In addition, a whole brain analysis was conducted on a voxelwise basis. The models used for the whole brain analysis were identical to those used in the ROI-based mixed effects models but simply conducted at the voxel level. Bonferroni (restricted to gray matter voxels) and non-parametric methods of thresholding (N=500 random permutations of QUIC scores thresholded at p<0.01 and testing for likelihood of clusters larger than right amygdala cluster) were used for multiple comparisons correction. Qualitative inspection supported significant *uncorrected* clusters bi-laterally in the amygdala, with the peak and extent of whole amygdala coverage greater in the right amygdala (Supplementary Figure S1). While anatomical specificty of effects modestly supports localization to the amygdala and hippocampal head, the magnitude of effects was not significant at a Bonferroni threshold nor were they significant using non-parametric methods for thresholding. In part, this may be as expected based on the anatomical constraints of the size of the amygdala available for cluster volume compared to the cortex and the expected effect size between exposure (unpredictability) and outcome (task-based activation).

Psychophysiological Interaction (PPI) analyses were centered on the right amygdala based on the following rationale. First, PPI analyses necessitate identifying a lateral seed region

because bilateral averaging of spatially separated time courses may obscure important frequency characteristics used for connectivity measurement. Second, considering each lateral ROI would result in n=15 ([(N_{ROI}-1)*N_{ROI}]/2) comparisons, requiring what is likely to be an implausible effect size for multiple comparisons correction. Third, theoretical considerations support a focus on the right amygdala with broader evidence supporting lateral specialization in rapid and unconscious emotional processing (5), novel relative to familiar contexts (6), and in the context of ELA (4). Thus, in consideration of the above and our observation of the strongest effects in the right amygdala by both magnitude and extent (see Supplementary Figure 1), we chose the right amygdala for analyses aimed at understanding condition-specific signal correlations within a lateral limbic network (amygdala, hippocampal head, hippocampal tail/body).

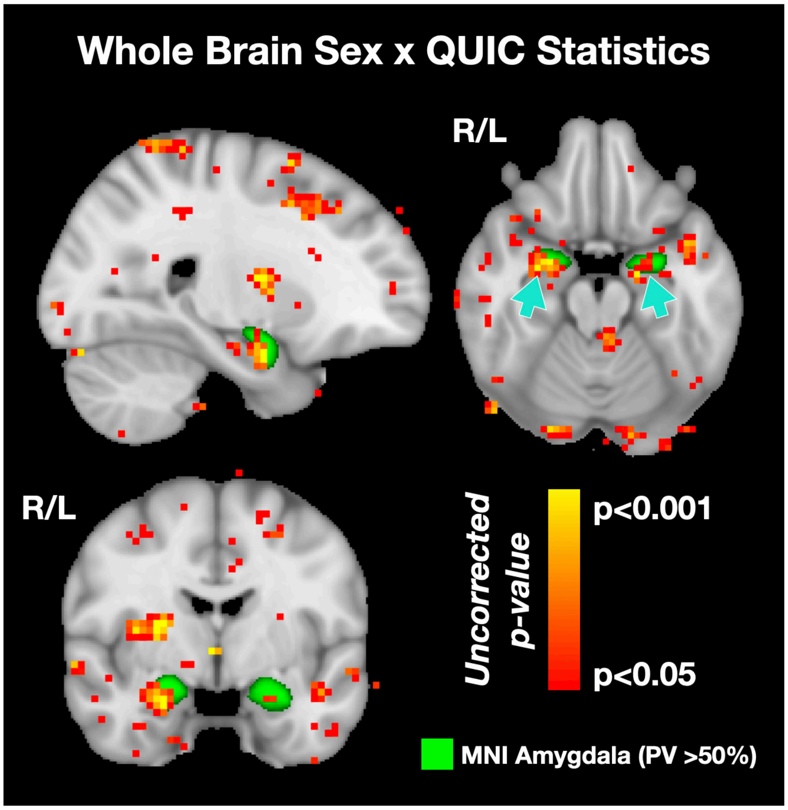

Supplementary Figure 1. Whole Brain Statistics. A whole brain parameter map (p-value: QUIC x sex interaction; thresholded at $p<0.05$) supports localization in the amygdala bi-laterally with a qualitatively stronger (peak and extent) effect in the right compared to the left. Note that no clusters survived non-parametric multiple comparisons correction.

	Left		Right		Bi-lateral	
	**t**	**p**	**t**	**p**	**t**	**p**
**Amygdala**	*2.24*	*0.026*	*2.42*	*0.015*	*2.69*	*0.007*
**Hippocampal Head**	1.44	0.152	*2.19*	*0.029*	*2.00*	*0.047*
**Hippocampal Body/Tail**	0.44	0.657	1.02	0.310	0.74	0.460

Supplementary Table 1. Lateralized Model Statistics. Six additional independent models were conducted to test for a laterality of effects. Evidence supports a rightward asymmetry and localizes the amygdala as having the strongest effect. Bi-lateral statistics (right columns) are redundant with those reported in the main manuscript but provided for reference.

Supplement S3: Sex-Specific QUIC Associations in the Amygdala Are Robust to Age Distribution and Modeling Considerations in the Context of Participant Age

The main manuscript used a mixed-effects model to account for sampling heterogeneity and spread in ages at visit. Given that late childhood/early adolescence is a period of rapid development, we sought to understand the influence of sample selection and modeling considerations on the statistical findings. Specifically, we considered three additional approaches: 1) considering the oldest visit only, 2) aggregating across visits by averaging variables, and 3) repeating analyses in a narrower age band (10-13 years old). Broadly, we found that the primary sex-specific associations with QUIC are robust to age considerations in the amygdala. Specifically, this is demonstrated through continued statistical significance (despite the reduced sample size) and/or a persistent effect size and direction.

	**n (observations)**	**t QUIC x Sex**	**p QUIC x Sex**
Bi-lateral Amygdala			
**Reference**	n=150 (223)	*2.69*	*0.007*
**Oldest Visit**	n=150 (150)	1.76	0.080
**Averaged**	n=150 (150)	*2.42*	*0.017*
**Narrow Range**	n=105 (107)	*2.06*	*0.041*
Bi-lateral Hippocampal Head			
**Reference**	n=150 (223)	*2.00*	*0.047*
**Oldest Visit**	n=150 (150)	1.56	0.12
**Averaged**	n=150 (150)	1.81	0.07
**Narrow Range**	n=105 (107)	1.12	0.27
Bi-lateral Hippocampal Body/Tail			
**Reference**	n=150 (223)	0.74	0.460
**Oldest Visit**	n=150 (150)	1.01	0.315
**Averaged**	n=150 (150)	0.61	0.544
**Narrow Range**	n=105 (107)	-0.08	0.929

Supplementary Table 2. Models Repeated Under Different Sampling and Modeling Strategies. Primary sex-specific associations with QUIC were largely robust to sampling and modeling strategies in the context of participant age.

Supplement S4: Supplement to Accompany Main Figure 3

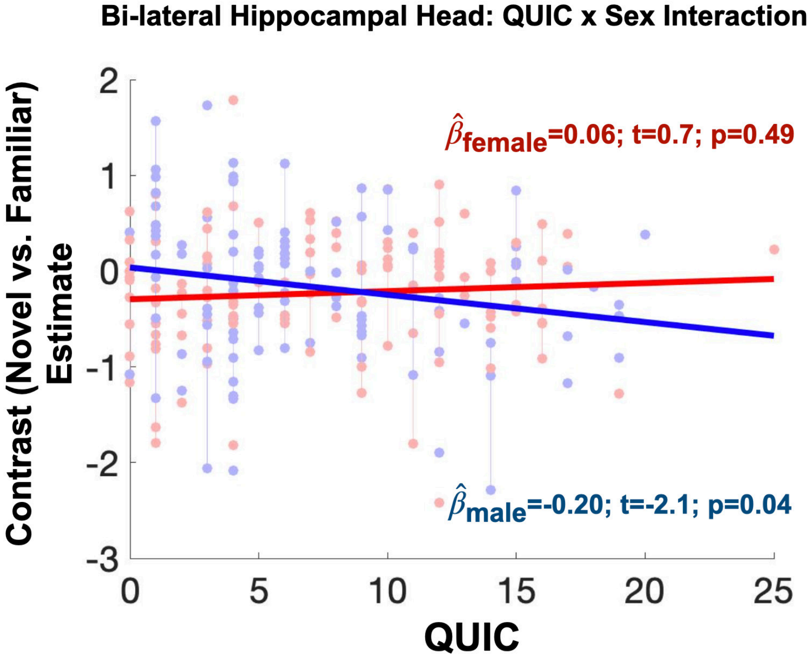

Figure S2. Early Life Unpredictability (QUIC) Is Associated with Hippocampal Head Activation to Novel vs. Familiar Scenes in a Sex-Specific Manner. Scatter plot depicting sex-specific associations.

Supplement S5: Supplement to Accompany Main Figure 4

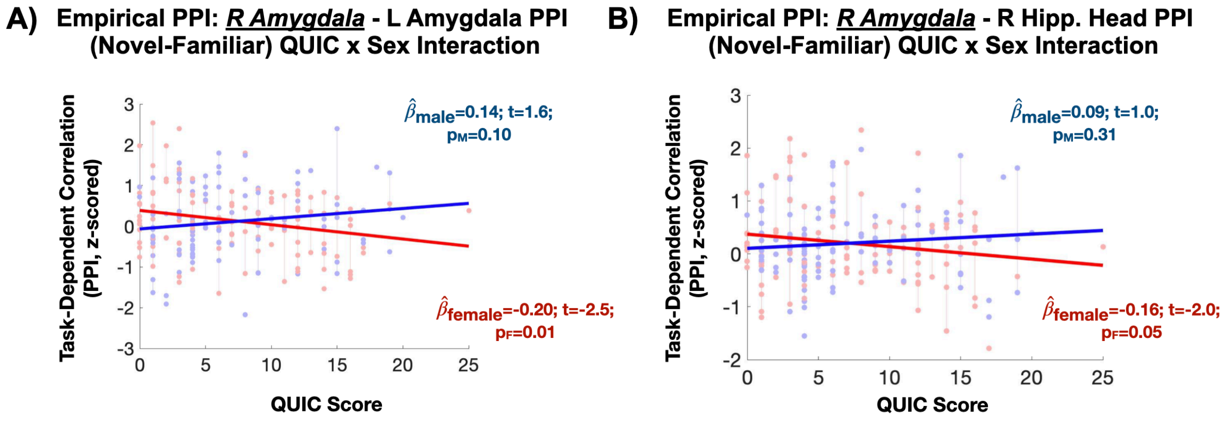

Figure S3. Early Life Unpredictability Is Associated with Task-Based Functional Connectivity of the Right Amygdala in a Sex-Specific Manner. Empirical scatter plot showing sex-specific associations between early-life unpredictability (QUIC) and PPI-based task-dependent correlations between the right amygdala and left amygdala, and right hippocampal head.

1. Glynn LM, Stern HS, Howland MA, Risbrough VB, Baker DG, Nievergelt CM, et al. Measuring novel antecedents of mental illness: the Questionnaire of Unpredictability in Childhood. Neuropsychopharmacology. 2019;44(5):876-82.
2. Davis EP, Glynn LM. The Power of Predictability: Patterns of Signals in Early Life Shape Neurodevelopment and Mental Health Trajectories. Journal of Child Psychology and Psychiatry. 2024.
3. Hunt C, Vinograd M, Glynn LM, Davis EP, Baram TZ, Stern HS, et al. Childhood unpredictability is associated with increased risk for long- and short-term depression and anhedonia symptoms following combat deployment. . J Mood Anxiety Disord. 2024.
4. Teicher MH, Khan A. Childhood Maltreatment, Cortical and Amygdala Morphometry, Functional Connectivity, Laterality, and Psychopathology. Child Maltreat. 2019;24(4):458-65.
5. Ocklenburg S, Peterburs J, Mundorf A. Hemispheric asymmetries in the amygdala: A comparative primer. Prog Neurobiol. 2022;214:102283.
6. Molodtsova GF. [Sexual and interhemispheric differences in the involvement of serotonin from the hippocampus and amygdaloid body in the processing of new and repeatedly presented information in rats]. Zh Vyssh Nerv Deiat Im I P Pavlova. 1999;49(3):408-15.
